# Dr. B. Wood on Dental Education

**Published:** 1853-01

**Authors:** 


*Dr. B. Wood on Dental Education.*—The conclusion of Dr. Wood's article on dental education will be found in the present number of the Journal. The article is well written, but we fear the doctor has not given the subject as much careful thought as its importance demands. We had occasion, a few months since, to notice a series of articles from his pen "on the propriety of establishing a college of dental surgery in Nashville," published in a paper of that place, and in which, the importance of such an institution there, is strongly urged. This recommendation not having been carried out, he soon after suggested the idea of uniting faculties of dentists to the faculties of medical colleges, offering as a reason for such union, that dental colleges could not furnish the necessary amount of medical instruction. Surely their means of instruction are no less ample now than they would have been had a college of dental surgery been established at Nashville. The last suggestion not having been any more favorably received than the first, he now contends strenuously for a preliminary medical course, but we fear that his zeal has led him into injustice towards dental colleges—the value of which to the student of dental surgery he so ably set forth a few months since, and which it must be acknowledged, have done more than any other single influence to advance the profession to its present position. *Jus cuique suum tribure* is a good motto for all. When the profession demands of dental colleges a higher standard of education, it should remember, with some slight gratitude, how much it is indebted to these institutions for the very feeling which prompts such a demand.

As an instance of injustice, we cite the calculation in page 36, which reduces the whole sum of medical instruction given in the Baltimore College to two, and in very many cases *one* month. He omits altogether the very important preliminary month devoted to the infirmary and dissections; and loses sight of the fact that out of the two classes of *one* term candidates, to wit, graduates in medicine and dental practitioners—each will give the larger portion of their time to that in which they are most deficient. The graduate in medicine will apply himself to the specialty of dentistry, while the dentist can secure his full quota of two months medical instruction. But two months can no more give the dentist his full share of medical knowledge, than can two months study of surgery make the medical student a perfect surgeon. Our colleges, dental or medical, do not claim to be omnipotent Jupiters, from which, Minerva like, springs forth young men, armed cap à pied and of full grown stature. A course of four times two winters, would fail to bring forth such prodigies. What our colleges profess to do for the faithful student is to prepare him to *commence* practice. The Baltimore College does this as effectually for the dentist as any medical college in our country for the young physician. What proves the one insufficient by parity of reasoning involves the other. Would the doctor add another term to the dental course, or require a preliminary medical course. Then he must think the education of the thousand physicians who have gone forth from our first medical colleges wofully deficient, unless, indeed, he adopt the rather untenable position that the dentist requires the more thorough training. Would he urge that a medical education is an essential prerequisite to a course of dental study? When he *proves* this, then we will undertake to prove that collegiate education must necessarily precede either. Doubtless the M. D., if well earned, would be a most desirable antecedent to the D. D. S.; but equally so the A. B. or A. M. They are neither of them so far indispensable as to warrant dental colleges in demanding them as a condition of matriculation.

The general principles of medicine are taught in both dental and medical schools, but they must of necessity vary in the special applications thereof. Dental students are taught the diseases of childhood, and the influence of pregnancy upon the general economy, for these have frequently a close relation to the teeth; but they are not taught obstetrics, for the sufficient reason that they will never be called upon to practice midwifery. They are taught how to excise the lower jaw, to tie the carotid artery, cure hair lip and congenital fissure of the palate; but they are not taught the details of the operation for strangulated hernia. This is not in the line of their profession, and, however desirable such knowledge is in itself, a dental college is neither bound to require or to teach it. Until Dr. Wood shall convince us to the contrary we shall continue in the belief that the Baltimore College teaches all the branches, medical and surgical, necessary to qualify her graduates to commence the practice of dental surgery.

We may renew the consideration of this subject in our next number.

